# Sialic Acid Receptors: The Key to Solving the Enigma of Zoonotic Virus Spillover

**DOI:** 10.3390/v13020262

**Published:** 2021-02-08

**Authors:** Suresh V Kuchipudi, Rahul K Nelli, Abhinay Gontu, Rashmi Satyakumar, Meera Surendran Nair, Murugan Subbiah

**Affiliations:** 1 Animal Diagnostic Laboratory, Department of Veterinary and Biomedical Sciences, Pennsylvania State University, University Park, PA 16801, USA; abhinay@psu.edu (A.G.); rks5672@psu.edu (R.S.); mms7306@psu.edu (M.S.N.); 2 Center for Infectious Disease Dynamics, Pennsylvania State University, University Park, PA 16801, USA; 3 Veterinary Diagnostic Laboratory, College of Veterinary Medicine, Iowa State University, 1850 Christensen Drive, Ames, IA 50011, USA; rknelli@iastate.edu; 4 Molecular Biology Division, Maryland Department of Health, Baltimore, MD 21201, USA; murugan.subbiah@maryland.gov

**Keywords:** sialic acids, receptors, viral entry, zoonotic spill over, cross-species transmission, emerging viruses, pandemics

## Abstract

Emerging viral diseases are a major threat to global health, and nearly two-thirds of emerging human infectious diseases are zoonotic. Most of the human epidemics and pandemics were caused by the spillover of viruses from wild mammals. Viruses that infect humans and a wide range of animals have historically caused devastating epidemics and pandemics. An in-depth understanding of the mechanisms of viral emergence and zoonotic spillover is still lacking. Receptors are major determinants of host susceptibility to viruses. Animal species sharing host cell receptors that support the binding of multiple viruses can play a key role in virus spillover and the emergence of novel viruses and their variants. Sialic acids (SAs), which are linked to glycoproteins and ganglioside serve as receptors for several human and animal viruses. In particular, influenza and coronaviruses, which represent two of the most important zoonotic threats, use SAs as cellular entry receptors. This is a comprehensive review of our current knowledge of SA receptor distribution among animal species and the range of viruses that use SAs as receptors. SA receptor tropism and the predicted natural susceptibility to viruses can inform targeted surveillance of domestic and wild animals to prevent the future emergence of zoonotic viruses.

## 1. Background

The coronavirus disease 2019 (COVID-19) pandemic highlighted yet again the devastating global impact of emerging viral infections. A major aspect that contributes to viral emergence is the perpetual spread of animal viruses into human populations. Consequently, a major proportion of newly emerging viral infections are zoonotic in origin, those that transmit from animals to humans and vice versa [1]. RNA viruses in particular continue to emerge due to their high rates of nucleotide substitution and poor mutation error-correction ability [2]. Therefore, a thorough understanding of the risk factors for viral transmission at the animal–human interface is critical for disease prevention and outbreak containment [3].

A central question in understanding what drives virus epidemics and pandemics is why and how likely are viruses to spill over from one host to another. A key factor that determines the host tropism of viruses is their ability to bind with high affinity to specific host cell receptors. Viruses use their proteins to structurally mimic host ligands to interact with host cells [4]. To better predict and prevent future epidemics and pandemics, a key question that need to be answered is how various animal species manage to maintain infection as reservoirs, and thereby enable spillover to humans. Due to their widespread distribution on surface of many different cell types, across multiple species, sialic acids (SAs) play crucial roles in mediating attachment and entry to viruses belonging to many different families.

## 2. Sialic Acids and Their Biological Significance

Glycans are structural polysaccharides found ubiquitously on eukaryotic and prokaryotic cells and contribute to the cellular protective, stabilizing, organizational, and barrier functions [5]. The glycan chains of a majority of host cell glycoconjugates are composed of five- to six-carbon sugars. Sialic acids (SAs) are a striking exception to the family of sugar units with a nine-carbon backbone and typically found attached to the terminal position of N- and O-linked glycans [6]. The unique location of these nine-carbon alpha-keto acids helps them play a critical role in several intrinsic and extrinsic interactions of a cell [5,6,7].

Over the past decade, our understanding of SAs, including their diversity and function, has significantly increased [6,7]. Earlier, SAs were defined merely as neuraminic acid (5-amino3,5-dideoxy-D-glycero-D-galacto-2-nonulosonic acid; Neu) and its derivatives [8,9]. The definition was expanded in the late 1900s, with the discovery of 3-deoxy-D-glycero-D-galacto-2-nonulosonic acid (or 2-keto-3-deoxy-D-glycero-D-galacto-nonionic acid; KDN), which has a hydroxyl group in place of the amino group at the C5 position [10]. Currently, in nature, more than 50 structural variations for SAs have been elucidated, and each of which are known to be attached to multiple glycans using different types of α-linkages from the C2 position [11]. Numerous substitutions at the 4, 5, 7, 8, and 9 positions along with the linkage variations also added to the rare diversity of SAs [7,11,12]. The most common mammalian SAs are C5 structural variants with either an acetamido, hydroxyacetamido, or hydroxyl moiety to form 5-N-acetylneuraminic acid (Neu5Ac), 5-N-glycolylneur-aminic acid (Neu5Gc) or deaminoneuraminic acids (KDN), respectively [12].

The remarkable diversity of SAs and their unique chemical and physical properties, including hydrophilicity and intrinsic negative charge, are reflected in the wide array of their biological roles [13,14] ranging from immune responses [15] to nutrition [16], development, as well as renal and neural functions [14,17,18]. SAs have also been implicated in various pathophysiological processes such as oncogenesis [13] and microbial pathogenesis [17,19].

## 3. Sialic Acid Receptors of Emerging and Zoonotic Viruses

Many viruses across virus families often target particular classes of receptors, such as sialylated glycans, cell adhesion molecules, to mediate cellular entry. The redundancy in receptor usage indicates evolutionary conservation in the way viruses target particular receptors to take advantage of their cellular function [4].

Sialo-glycoconjugates expressed on cell surfaces serve as ligands or receptors for intrinsic or extrinsic SA specific lectins. Often, microbes express the SA-specific lectins to decorate their cell surfaces to aid in host cell attachment and assist in the evasion of host immunity [10]. Many RNA viruses and DNA viruses exploit these glycans as initial anchors to gain access to the host cells. Host cell receptors undergo evolutionary alterations to avoid rapidly emerging pathogens while maintaining the critical endogenous function. Consequently, many of the microbial interactions with host cells expressing cognate SA are detrimental. Most viruses have evolved to express enzymes that can cleave the interactions with these SA receptors, aiding their release from infected host cells. These sialidases act as decoy receptors, which bind to virions, preventing their access to host epithelial cells [18].

Although recent developments in the field of glycan biology have aided in the knowledge advancements of such different structural variants of N- and O- and glycosphingolipid-glycans present in the respiratory tract of human and other host species, little is known about the intricated interactions among different endogenous and exogenous SA-specific ligands in fighting pathogenic viruses by modulating immune tolerance. The following sections detail the distribution profile of SA receptors in different hosts and their potential influence in host jumps and pathogenesis.

## 4. Distribution of Sialic Acid Receptors among Multiple Host Species

**Figure 1:**
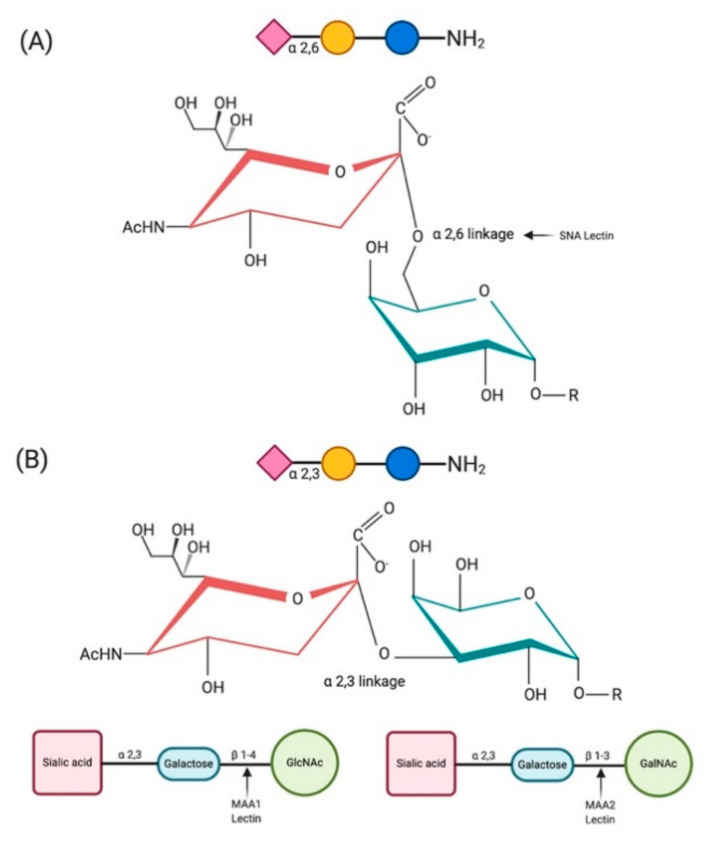
Schematic illustration of SAα2,6-Gal (**A**) and SAα2,3-Gal (**B**) receptors highlighting the binding pattern of linkage-specific lectins.

Sialic acids have conserved biochemical structures and are widespread all across the vertebrate hosts, offering attractive attachment sites for viruses. Various methods have been used to characterize the SA receptor distribution profiles among multiple host species. The presence of appropriate an receptor for a virus was initially studied using virus-binding assays with extracted total gangliosides from cell membranes [20]. Later, lectin histochemistry using linkage-specific lectins, which can selectively recognize the sialic acid linkage to sugars, were used. For example, *Maackia amurensis* (MAA) lectins MAA I and MAA II were used to detect α2,3-linked SA, and *Sambucus nigra* (SNA) lectin or *Sambucus sieboldiana* agglutinin (SSA) was used to detect α2,6-linked SA [21,22,23,24]. It was found that the MAA lectins are specific to the third sugar residue of the SAα2,3-Gal receptors. MAA I and MAA II are specific for SAα2,3-Galβ(1-4)GlcNAc and SAα2,3-Galβ(1-3)GalNAc, respectively [25] (Figure 1).

Mass spectrometry or MALDI-TOF analyses are also used to identify the SA receptor distribution in the tissues [26,27,28]. More recently, glycan microarrays have been employed to study the distribution of SAs as well as the functional aspects of glycan–protein interactions [29,30,31].

The presence or absence of an appropriate host SA receptor is a major determinant of the host tropism of a virus including the tissue and the specific cell types within the host that the virus can infect [18]. Host receptor distribution is therefore a key to understand their susceptibility to a particular virus and to determine the body systems that will likely be infected and the type of clinical symptoms. The viral protein binding to cells of various tissues play a crucial role in the spread of viral infection throughout the body of the host [32,33]. For example, H5N1 avian influenza virus was shown to spread to the central nervous system because of the preferential binding of the virus to α2,3-SAs in the nervous system [33]. The distribution of SA variants in various tissues and hosts may contribute to the evolution of specific viral glycoproteins, which can interact with SAs [18,34]. Several viral glycoproteins have also been found to interact with SAs on the erythrocytes of various species, resulting in their agglutination. The ability of viruses to cause hemagglutination has been extensively used in clinical diagnosis of viral infections [35]. The expression and distribution of these SA receptors differ depending on the location within the body, type of the cell, and their intended functional role (Table 1).

### 4.1. Humans

Humans predominantly express SA α2,6-Gal receptors in the ciliated and non-ciliated epithelium of the respiratory tract, extending from the paranasal sinus to alveolar cells [25,33,36,37]. SA α2,3-Gal receptors are found in the ciliated epithelium lining the bronchioles and alveoli [30,38]. Age-dependent differences in the distribution of SA receptors have been reported in humans. SNA binding, used to detect α2,6-linked SA, was found to be stronger in the epithelium from children compared to adults [22]. Widespread and stronger binding to MAA I was found in the upper and lower respiratory tracts in pediatric epithelium compared with adults, while the binding to MAA II is restricted to alveolar epithelium with a weak binding to goblet cells [22]. The distribution of sialic acid receptors requires further research, with recent reports suggesting the presence of SA α2,6-Gal receptors in the ileal epithelium [39], in SA α2,3-Gal receptors in the colon epithelium [40], on the endothelial cells of blood vessels, and on inflammatory cells [36].

### 4.2. Non-Human Primates

Non-human primates diverge from their human counterparts in their expression pattern of SA receptors. N-acetylneuraminic acid (Neu5Ac) and N-glycolylneuraminic acid (Neu5Gc) are the two major sialic acid receptors found on most mammalian cell surfaces. Humans evolutionarily carry a mutation in CMP-Neu5Ac hydroxylase and, as a consequence, lack Neu5Gc and diverge from the non-human primate ancestors [37]. Chimpanzees, gorillas, and orangutans express SA α2,6-Gal receptors abundantly in goblet cells but lack their expression on the epithelial cells of the trachea and the lung [22,41]. In African green monkeys, SA α2,6-Gal receptors are expressed along the epithelium of ciliated cells in the upper respiratory tract (URT), extending to the vascular endothelial cells of lungs. SA α2,3-Gal receptors are expressed by type II pneumocytes in the lung [42], while both SA α2,6-Gal and SA α2,3-Gal receptors are expressed in the goblet cells of the submucosal glands and submucosal connective tissue [42].

### 4.3. Livestock Species and Farm Animals

#### 4.3.1. Swine (Pigs)

In pigs, SA α2,6-Gal receptors are dominant in the ciliated pseudostratified epithelia and goblet cells along the respiratory lining of trachea and bronchus [43]. The alveolar epithelial lining expresses both SA α2,6-Gal and SA α2,3-Gal receptors with a relative rise in the expression of SA α2,3-Gal receptor from the upper respiratory tract (sub-epithelial locations) to the lung epithelium [43]. Similarly, widespread distribution of α2,3-SA and α2,6-SA was found in both the lung and the bronchus [31]. One study reported that MAA I binding was much higher compared to the MAA II binding in the epithelial lining of the bronchioles and alveoli of pigs [43], whereas another study reported the absence of MAAII binding in the pig lungs [44]. In the small intestine (duodenum), both SA α2,3-Gal and SA α2,6-Gal receptor types have been detected, with the SA α2,6-Gal receptor being the dominant receptor. It is localized along the epithelial border and in goblet cells [43]. The large intestine (colon) of the pig strongly expresses both SA α2,3-Gal and SA α2,6-Gal receptors on the epithelial border and in the goblet cells, where co-expression is frequently observed [43]. Neu5Gc is reportedly present in the skeletal muscles, lungs, spleen, kidneys, heart, and liver in pigs [45]. The discrepancy in the SA distribution pattern among different studies could be due to the methodology used and the potential differences in the glycan phenotypes among various breeds of swine [44].

#### 4.3.2. Equines (Horses)

The expression of both SA α2,6-Gal and SA α2,3-Gal receptors was recorded from nasal mucosa to bronchus and in goblet cells. SA α2,3-Gal is predominantly expressed on the surface of ciliated epithelial cells of the nasal mucosa, trachea, and bronchus, whereas SAα2,6 Gal is confined to cilia [46].

#### 4.3.3. Bovines (Cattle)

The trachea of cows is deficient in both SA α2,6-Gal and SA α2,3-Gal receptors. Even water buffaloes lack SA α2,6-Gal receptors in their tracheal epithelium, and intriguingly they still exhibit SA α2,3-Gal receptors in the trachea [47]. SA 9-O-Ac, another sialic acid receptor that plays a significant role in influenza D virus infection, is found in the respiratory tract of cows [48]. High levels of 9-O-Ac SA receptors are also observed in bovine submaxillary mucin [49]. Neu5Gc is a commonly found sialic acid receptor in the skeletal muscle and organs in cattle [45]. However, more studies are needed to understand the distribution of the different SA receptors in bovine species.

#### 4.3.4. Camelidae (Camels)

The apical side of the nasal respiratory epithelium and the sub-epithelial regions of camels express SA α2,3-Gal. These receptors are also abundant in the secretory goblet cells of the nasal epithelium. In the lungs, SA α2,3-Gal receptors are abundant in alveolar epithelial cells [50]. The expression patterns of SA α2,6-Gal receptors in respiratory tract tissues have not been reported till date.

### 4.4. Companion Animals

#### 4.4.1. Canines (Dogs)

In dogs, SA α2,3-Gal receptors are expressed from the surface of ciliated epithelial cells of the nasal mucosa, continuing into the trachea and bronchi, up to alveoli of the lung [51]. Both SA α2,3-Gal and SA α2,6-Gal receptors are present in the goblet cells and sub-epithelial regions of nasal mucosa and trachea, but the SA α2,6-Gal expression is relatively weaker [52]. SA α2,6 Gal expression is mainly distributed in goblet cells, lamina propria, and submucosal layers [52]. The large intestine also expresses both SA receptors, with the expression of SA α2,3-Gal receptors being more predominant [52]. Breed-specific differences in SA receptor distribution among dog breeds have been reported [52]. However, it is not entirely clear if these are real breed-specific differences or differences due to the source of lectins used in these studies and therefore need further investigation.

#### 4.4.2. Felines (Cats)

The ciliated pseudostratified columnar epithelial cells and goblet cells of cats exhibit both SA α2,3-Gal and SA α2,6-Gal receptors. The expression of SA α2,6-Gal receptors is enhanced in the goblets cells [53]. Although SA α2,3-Gal are absent in the tracheal cranial epithelium, their expression was noticed in sub-epithelial connective tissue and in a small proportion of goblet cells [53]. Co-expression of both SA α2,6-Gal and SA α2,3-Gal receptors was observed on alveoli epithelial cells, with SA α2,6-Gal being predominant [51].

### 4.5. Wild Animals

#### 4.5.1. Bats (Order: Chiroptera)

Bats are mammals of the order Chiroptera in the kingdom Animalia, composed of a large number of diverse species. A few studies have reported the SA expression in some species. Little brown bats (*Myotis lucifugus*) are shown to have SA α2,3-Gal receptor as a predominant receptor type on the respiratory epithelium of trachea. It is also a predominant receptor type on the mucosal lining of the intestinal villi [54]. Although SA α2,6-Gal receptors are limited to lamina propria and submucosa of the trachea and bronchi, their expression is dominant in the alveoli of the lung [54]. In intestines, SA α2,6-Gal receptors are more prominent in the goblet cells, lamina propria, muscularis, and serosa of the intestine [54]. The characterization of lung cell lines of bats (*Tadarida brasiliensis*) also revealed extensive SA α2,3-Gal receptors with barely detectable SA α2,6-Gal receptors (Table 1) [55].

#### 4.5.2. Plateau Pika (*Ochotona curzoniae*)

Widespread distribution of SAα2,6Gal receptors across many tissues and organs has been reported in wild plateau pika. Notably, SA2,3Gal-linked receptors were found to be the dominant receptor type on the tracheal epithelial cells. Both SAα2,3Gal and SAα2,6Gal receptors are co-expressed in the lamina propria and mucous glands of the trachea, whereas SAα2,6Gal receptors are predominant in the alveolar cells. Both SA α2,6-Gal and SA α2,3-Gal receptors are expressed in the duodenum, ileum, and rectum. The epithelial lining of the intestinal mucosa, goblet cell, and lacteals exhibit the presence of both the receptors, but SA α2,3-Gal expression is predominant on the intestinal epithelium [57].

#### 4.5.3. Raccoon (*Procyon lotor*)

The predominant receptor on the epithelium and sub-epithelial regions in the URT of the raccoon is SA α2,6-Gal. The expression of SA α2,3-Gal receptors gradually increase in both epithelial and sub-epithelial regions of the trachea and bronchi. Similar expression patterns of both receptors have been reported in the lungs [58].

### 4.6. Laboratory Animals

#### 4.6.1. Guinea Pig (*Cavia porcellus*)

Expression of both SA α2,3-Gal and SA α2,6-Gal receptors is observed on the epithelial cells of nasal and tracheal mucosa. However, in the lungs, SA α2,3-Gal is the dominant receptor type in alveolar cells and the vascular endothelial cells [59].

#### 4.6.2. Ferrets (*Mustela putorius furo*)

Ferrets are widely used as animal models for the study of influenza A infection because they have a similar distribution of SA α2,3-Gal and SA α2,6-Gal receptors in the respiratory tract as humans [60,61]. SA α2,6-Gal is expressed abundantly by ciliated cells and submucosal glands in the trachea and bronchus of the ferrets. SA α2,3-Gal is expressed in lamina propria and sub-mucosal areas. Both SA α2,3-Gal and SA α2,6-Gal are found in the alveoli, although SA α2,6-Gal is more abundantly expressed [53].

#### 4.6.3. Hamsters (*Mesocricetus auratus*)

Hamsters are similar to ferrets in their distribution of sialic acid receptors and, hence, are popularly used as animal models for influenza studies. SA α2,6-Gal is expressed on ciliated cells, while SA α2,3-Gal receptors are found on both ciliated and non-ciliated cells [62]. A recent study reported that SA α2,6-Gal are expressed in the distal end of the nasal cavity, while SA α2,3-Gal are expressed in the An section. Both receptors are present in the pharynx, trachea, and bronchus, and only SA α2,3-Gal is found in the lungs [63].

#### 4.6.4. Mice (*Mus musculus*)

The nasal cavity of BL6 mice lack SA α2,6-Gal receptors and SA α2,3-Gal receptors on the non-ciliated epithelial cells, but the sub-epithelial locations are rich in both receptors. However, their expression has been reported on both ciliated and non-ciliated cells of the trachea, bronchi, and bronchioles [64]. Co-expression of both SA α2,6-Gal and SA α2,3-Gal was reported in the alveolar cells of the lung [64]. Mice intestines express both SA α2,6-Gal and SA α2,3-Gal receptors. The aggregated lymphoid nodules in the connective tissue of cecum are noted to be rich in SA α2,6-Gal receptors [64].

### 4.7. Terrestrial Birds

#### 4.7.1. Galliformes

The birds that are most frequently encountered and domesticated by humans are chickens, turkeys, pheasants, partridges, grouse, quails, and guinea fowls. These birds typically express both SA α2,6-Gal and SA α2,3-Gal receptors from the nasal cavity to the lungs, with varying degrees of expression in each region of the respiratory tract. For example, the tracheal epithelium of the chicken (*Gallus gallus*) and the quail (*Coturnix coturnix*) showed predominant SA α2,6-Gal receptors [29,67]. In chicken trachea, the dominant receptor type is SAα2,6-Gal [29], with SA α2,3-Gal receptors being the abundantly distributed receptor type in the intestines [65,67]. Further investigation of SAα2,3-Gal receptor subtypes in chickens revealed that both SAα2,3-Gal β(1-3 )GalNAc and SAα2,3-Gal β(1-4) GlcNAc receptor subtypes are detected in the sub-epithelial region of trachea. However, along the chicken tracheal epithelium, SAα2,3-Gal β(1-4) GlcNAc receptors were more dominant than SAα2,3-Gal β(1-3) GalNAc receptors [29].

#### 4.7.2. Passeriformes

This order comprises crows, ravens, sparrows, cuckoos, and other songbirds. The respiratory tract epithelial cells of these birds vastly express SA α2,3-Gal receptors, along with some SA α2,6-Gal expression. Predominant distribution of SAα2,3-Gal β(1-3)GalNAc receptor type was observed in the respiratory tract of most birds in this order [21]. The sub-epithelial regions and glands represent both SA α2,3-Gal and SA α2,6-Gal receptors, with occasional co-expression [21]. SA α2,3-Gal is the dominant receptor type in the intestines of these birds, and presence of both SAα2,3-Gal β(1-3)GalNAc and SAα2,3-Gal β(1-4)Glc NAc has been identified [21]. SA expression is reported to vary among various species of this order [66].

#### 4.7.3. Columbiformes

This order comprises pigeons and doves. The respiratory tracts of these birds are reported to be rich in both SA α2,6-Gal and SA α2,3-Gal receptors. The intestines of rock pigeons *(Columba livia)* continue to maintain SA α2,3-Gal receptors along their length, but fail to express SA α2,6-Gal receptors [21]. Of the type of SA α2,3-Gal receptors, SAα2,3-Gal β(1-4) GlcNAc receptors were reported to be dominant in these birds [21]. However, inter-species variations in the SA expression among the birds of this order have been reported [66].

### 4.8. Aquatic Birds

Understanding the sialic acid distribution in wild aquatic birds is of relevance because some of these birds are reservoirs of avian influenza.

#### 4.8.1. Anseriformes

The most commonly known species of the order Anseriformes are ducks, geese, and swans. These species show both SA α2,6-Gal and SA α2,3-Gal receptors in the respiratory and intestinal tracts. SA α2,3-Gal receptors are dominant on the respiratory epithelial cells from nasal turbinates all the way into the lungs of these birds [29,65]. These receptors are abundant in ciliated cells, goblet cells, and sub-epithelial regions of the trachea and bronchi, too [21]. Abundant binding of the two isoforms of MAA lectin, MAA I and MAA II, was observed in most birds of the order, with stronger binding found with MAA II [21]. The distribution of SA α2,6-Gal receptors is enhanced as we progress deeper into the lungs. In the intestinal tract of Anseriformes, again, SA α2,3-Gal is comparatively a dominant influenza virus (IAV) receptor type and is distributed in goblet cells across the small and large intestines. Although the ileal and cecal enterocytes are rich in SA α2,6-Gal receptors, the duodenum and jejunum have varying levels of SA α2,6-Gal expression [21].

#### 4.8.2. Charadriiformes

This group comprises seagulls and other sea birds such as puffins and guillemots. Seagulls (*Larus smithsonianus*, *Larus atricilla*, *Larus delawarensis*) are abundantly rich in both SA α2,6-Gal and SA α2,3-Gal receptors in the respiratory tract epithelium. However, the expression of SA α2,6-Gal is marginal in the intestines of gulls [21].

#### 4.8.3. Gruiformes, Pelecaniformes, Gaviiformes, Ciconiiformes

These orders include wild aquatic birds such as large cranes, pelicans, loons, and herons. These species are positive for SA α2,3-Gal receptors both in the respiratory and intestinal epithelium. Although SA α2,6-Gal receptors are expressed in respiratory epithelium, they are absent in the ileum and the caeca of these species [21]. Most of the species from these orders show binding to both isoforms of MAA lectins in the intestines, with predominance of MAA II binding in the respiratory tract [21].

While there are many reports describing the SA receptor distribution in the tissues of various hosts, the methodology used could have contributed to the observed discrepancies among these studies. The earlier analyses were done using extracted gangliosides from plasma membranes that were tested for virus binding [20]. The limitation of such analyses is that they cannot capture the normal anatomical distribution of the SAs on mucosal surfaces. Lectin histochemistry on tissue sections was more suitable for detecting the distribution patterns and relative abundance of SAs on the mucosal lining of the respiratory and gastrointestinal systems. However, it was subsequently found that lectins from various suppliers differed in their specificities [22]. It was shown that using both MAA I and MAA II lectins to probe SA α2,3-Gal receptors is crucial as these lectins actually detect the linkage to the third sugar residue [29]. In addition, methods used to visualize the lectin binding such as chemical and fluorescent detection have varying sensitivities. Even with fluorescent detection, some studies have used ordinary fluorescent microscopy, whereas other studies have used confocal microscopy, which could also have contributed to the discrepancies [29]. Therefore, development of a standard and reproducible method is important so that all future studies investigating SA receptor distribution can be comparable.

As we discussed the SA receptor distribution among various hosts, it is imperative to understand how the susceptibility of different host species to viruses varies based on this distribution profile. The following section will discuss the potential implication or role of SA as receptor determinants for the pathogenesis of various virus families and how distribution profile affects the emergence and re-emergence of viral pathogens in various hosts.

## 5. SAs as Receptor Determinants for Mammalian Viruses

### 5.1. RNA Viruses

#### 5.1.1. Orthomyxoviridae

Influenza viruses (IAVs) are a major group of zoonotic viruses that cause several human pandemics and continue to pose a very high risk. All the past influenza pandemics were caused by IAVs that emerged either entirely or partially from animal reservoirs. Influenza A viruses can be broadly categorized as avian and mammalian viruses, based on their host range [68]. Waterfowl and shorebirds are the natural reservoirs for IAVs and contain 16 of the total 18 known strains of the virus [69].

The susceptibility of a host to IAV infection is determined by the type of SA receptor present on the host cell surface along with other host factors. Human and swine IAVs preferentially bind to SA linked to galactose via α2,6 linkage, while the HA of avian and equine IAVs preferentially bind to SA linked to galactose via α2,3 linkage [70,71,72]. The preferences of the sialic-galactose linkage type indeed correlate with the distribution of the SA receptors in the infection sites of the specific hosts and natural routes of transmission [20,73].

Mutations in HA that can change the binding preference can lead to cross-species transmission of IAVs. For example, the specificity of the avian influenza viruses (AIVs) of H2 and H3 subtypes shifted from avian type binding to α2,3 SA to that of human type α2,6 linked SA due to two mutations in glutamine-226-leucine and glycine-228-serine [74,75]. In the case of the 1918 pandemic, which involved the H1N1 strain of IAV, although the amino acids in their 226 and 228 positions were retained, structural variations were observed in the 220 amino acid loop of the HAs. The altered configuration lowered the position of glutamine 226 in the receptor and enabled HAs of H1 to bind to α2,6 linked SA in humans [76]. This binding was further strengthened by interactions between the amino acids in the 220 loop and H-bond between aspartic acid at the 190th position of HA and the SA receptors, enhancing the disease transmission [76,77]. The 2013 influenza outbreak, caused by the low-pathogenicity avian influenza (LPAI) H7N9 virus, the virus exhibited specificity to both α2,3 and α2,6 SA receptors, and the virus was found to attach to both the upper and lower respiratory tract of humans. Several studies involving mammalian models have indicated that H7N9 viruses were more pathogenic than typical human IAVs [78,79,80].

It is widely known that an excessive host cytokine response is responsible for the high pathogenicity of the highly pathogenic avian influenza (HPAI) H5N1 viruses in humans and chickens [81,82,83]. The hypercytokinemia seen in patients infected with HPAI H5N1 viruses is associated with high viral load, which leads to severe often fatal clinical outcome [84]. Notably, the H5N1 virus binding to SAα2,3 is sensed by human cells differently than the virus binding to SAα2,6, leading to an exacerbated innate pro-inflammatory response [85]. Till date, typical avian influenza strains have not been able to transmit among humans efficiently. However, the possibility of such an occurrence cannot be ruled out. Some recent studies indicate that not only H1, H2, and H3 but also H5, H7, and H9 can attain the ability of airborne transmission in mammalian hosts [75,77,86,87,88].

The HA of influenza B virus (IBV) can recognize both α-2,6- and α-2,3-linked SA residues and is attributed to the presence of phenylalanine at the HA amino acid position 95 instead of tyrosine being present in the same position in IAV [89]. Influenza C and D viruses (ICVs and IDVs) use membrane protein hemagglutinin-esterase fusion (HEF) to detect and bind to N-acetyl-9, O acetylneuraminic acid receptors [48]. The zoonotic potential of ICVs and IDVs is yet to be investigated [90,91,92,93,94,95]. Other orthomyxoviruses, such as the infectious salmon anemia (ISA) virus affecting fish, have a preferential binding toward 4-O-Acetylated SAs [96,97].

#### 5.1.2. Coronaviridae

Coronaviruses have caused three major human epidemics in the 21st century alone and continue to be a major threat to public health [98]. Coronaviruses infect a wide range of animals, birds, and humans and are capable of crossing the species barrier. Cross-species transmissions between cattle to humans is believed to have given rise to human coronavirus OC43 and human coronavirus 229E and, more recently, to canine respiratory coronavirus. Further research to investigate the possible contribution of SA receptors in facilitating the cross-species transmission is necessary.

There is increasing evidence that sialylated compounds of cellular glycocalyx can serve as an important factor in the mechanism of CoV infection [99]. With the ongoing SARS-CoV2 pandemic, there is at least in silico evidence that the sialic acid receptors can be potential entry receptors for the virus apart from the reported ACE2 receptors [100]. A study on glycosylation on the two subunits, S1 (facilitates attachment to host cell receptor via receptor binding domain) and S2 (mediates fusion of viral and human cellular membranes), of the viral spike protein S when it is expressed in human cells revealed unexpected O-glycosylation modifications in the receptor-binding domain of S1 [101]. It has been shown that the highly sialylated glycans present on specific sites on the receptor-binding domain can play a crucial role in viral binding with hACE2 [102,103,104]. The findings of a recent study on the glycosylation of hACE2 indicated the increased affinity of S protein to α-2,3- linked sialylated glycan at relatively higher levels. Therefore, α-2,3- linked sialylated glycans can facilitate the binding of S proteins of the coronavirus [105].

Previously, human coronavirus, MERS-CoV, was reported to recognize SA as a pre-attachment receptor, in addition to its cell entry receptor dipeptidyl peptidase 4 [105,106]. Recently, a study demonstrated that human coronaviruses OC43 and HKU1 bind to sialoglycan receptors and bind to its 9-0-acetylated SA as the primary binding component [107]. A subsequent study showed that the receptor-binding domain is conserved across coronaviruses to recognize terminal sialylated oligosaccharides present on the host cell surface [103].

Coronaviruses cause respiratory, enteric, and neural infections in several other mammals and birds [106] and known to interact with host cell SAs. The two porcine coronaviruses, transmissible gastroenteritis virus (TGEV), and porcine epidemic diarrhea virus (PEDV) were reported to have SA receptor binding ability. The point mutations on spike proteins of TGEV were shown to be associated with markedly lower viral pathogenicity owing to the abrogated sialic acid binding [108]. 5-N-acetylneuraminic acid (Neu5Ac), 5-N-glycolylneuraminic acid (Neu5Gc), and 5-N-acetyl-9-O-acetyl neuraminic acid (Neu5, 9Ac2) were recognized as co-receptors by the CoVs as their survival mechanisms are under unfavorable intestinal tract conditions [109,110].

As mentioned, betacoronoviruses, which include bovine coronavirus (BCoV) and porcine hemagglutinating encephalitis virus (PHEV), were observed to have a conserved hydrophobic pocket on the N-terminal domain of the spike protein, facilitating the interaction with N-acetyl-9- O-acetylneuraminic acid [103]. Interestingly, the 9-OAc-Sias-binding region on the spike protein shares structural similarity with CoV hemagglutinin esterase (HE) and ICV hemagglutinin esterase fusion (HEF) protein. Additionally, the avian CoV, infectious bronchitis (IBV) virus, recognizes the Neu5Gc as a binding receptor for infection and pathogenesis [111].

Furthermore, decades of study uncovering the molecular mechanisms underpinning the cell tropism and interspecies transmission has led to the discovery of the bivalent binding ability of CoVs. Many CoVs, including murine CoV- JHM and MERS-CoV, were shown to have spike proteins with two receptor-binding domains, an S1A that engages host sialic acids and an S1B that recognizes host transmembrane proteins. SA binding in CoVs enhances infection and promotes intercellular expansion through syncytial development [112]. Nevertheless, the role of sialic acid in tissue tropism and immune regulation in the CoV infections remains uninvestigated.

#### 5.1.3. Paramyxoviridae

Paramyxoviruses have been historically implicated in a multitude of zoonotic infections [113]. The strong epitope homology of the fusion protein of three viruses causing measles in humans, canine distemper in dogs, and rinderpest in bovines strongly suggests that these paramyxoviruses may have evolved from the same ancestor virus and post cross-species transmission have efficiently established in various hosts [114]. Hendra and Nipah, the two most deadly paramyxoviruses, are classical examples of zoonotic spillover in the not-so-distant past [115]. Several members of the Paramyxoviridae family of viruses bind to SA receptors on various host cells [116]. Paramyxoviruses—respirovirus, rubulavirus, and avulavirus—use viral fusion proteins, which can bind to SA cellular receptors, to gain their entry to the host system [117]. HPIV-3 is the most prevalent subtype of human parainfluenza viruses and preferentially recognizes α-2, 3- linked SAs in oligosaccharides present in the glycoprotein or glycolipids [116].

Mumps virus recognizes trisaccharide-containing α-2, 3- linked SAs present in unbranched sugar chains as the determinant receptor [118]. Although binding to receptors is not the only determinant for a cross-species transmission of a virus, a single amino acid change sometimes can shift the host tropism of paramyxoviruses. For example, canine distemper virus was experimentally shown to adapt to humans SLAM/F1 receptors with a single amino acid change [119]. Many novel paramyxoviruses have been identified from the surveillance of reservoir hosts whose receptors remain uncharacterized [120]. Investigating the receptor tropism of these novel paramyxoviruses is essential to evaluate their zoonotic potential.

#### 5.1.4. Flaviviridae

Flaviviruses are a major group of emerging and re-emerging arthropod-borne pathogens responsible for significant mortality and morbidity worldwide [121]. Flavivirus entry into the target cell is dependent on the envelope (E) protein contact with its cognate receptor. E protein initially binds to attachment factors such as glycosaminoglycans [121]. While the entry receptors for any flaviviruses are not yet fully identified [122], a wide range of cell surface receptors has been implicated in flavivirus entry [123]. The best characterized flavivirus entry receptors include α_v_β_3_ integrins, C-type lectin receptors (CLR), phosphatidylserine receptors TIM (T-cell immunoglobulin and mucin domain) and TYRO3, AXL, and MER (TAM) [121]. In addition to these receptors, the role of SA receptors in flavivirus cellular entry and pathogenesis is increasingly being recognized. Following the emergence of neurotropic Zika virus and its global spread, a study reported the role of α-2, 3- linked SA to facilitate the entry of Zika virus into host cells [122]. The role of SA receptors’ in the vector—virus transmission with the dengue virus—was also demonstrated [124]. Increased SA expression was associated with increased mortality and morbidity in a mouse model of the dengue virus, showing the dependency of the virus on SA receptors for causing potential pathogenesis [125]. These viruses have the potential to spread efficiently across the world as changing climate influences the transmission dynamics and geographic spread [126].

#### 5.1.5. Picornaviridae

Coxsackievirus A24 (CVA24), enterovirus 68 (EV68), and enterovirus 70 (EV70) variants of Picornaviridae are viruses of pandemic potential and use sialylated glycans as receptors to enter the host system [127,128,129]. EV68 causes mild to severe respiratory illnesses. CVA24v and EV70 have ocular tropism and are known to cause a highly contagious eye infection called acute hemorrhagic conjunctivitis [128,130]. CVA24 has been responsible for several outbreaks in the past decades [131]. EV70 binds Neu5Ac engaged in α-2, 3- linkages, while CVA24v binds to both α-2, 3- and α-2, 6- linked SAs with a higher preference to α-2, 6- linked SAs [129,132]. A glycan microarray experiment showed that EV68 can bind to SAs of both kinds of linkages but demonstrated a higher preference for α-2, 6- linked SAs [127]. Picornaviruses—enterovirus, parechovirus, and sapelovirus—of human origin have been shown to cause persistent infections or cycles of reinfection in non-human primates when housed in close proximity with humans, showing the potential for the occurrence of a spillback or a spillover event [133]. Of late, many rodent picornaviruses have been reported, which were associated with zoonoses [134,135]. Though not much is known, chickens are reported to be important reservoirs of a wide range of picornaviruses, which have the potential to cross the species barrier [136].

#### 5.1.6. Reoviridae

Reoviruses are another family of viruses that use sialylated glycans as co-receptors for initial interactions and entry into host cells. All reoviruses attach to junctional adhesion molecule-A (JAM-A), which is a component of intercellular tight junctions, with high affinity, and these are said to be the determinant receptors [137,138,139]. Melaka and Kampar viruses, belonging to the Orthoreoviridae family have been reported to cause acute influenza-like illness, after a species spillover from bats [140].

### 5.2. DNA viruses

#### 5.2.1. Adenoviridae

Adenoviruses are a common cause of ocular and respiratory infections in humans. SA-containing glycans act as the receptors for adenoviruses [141]. Evolutionary evidence is documented for cross-species transmission of adenoviral infections from various animal reservoirs to humans [142,143]. Further, novel adenoviruses have been reported to spread among humans originating from non-human primates and psittacine birds [144] with risks of a potential spread from bat adenoviruses [145].

#### 5.2.2. Parvoviridae

Parvoviruses are primarily known for their pathogenicity to animals, leading to hemorrhagic infections. Parvoviruses use glycan receptors for gaining access to the host cells, of which SAs are an important family [146]. Feline parvovirus evolving to canine parvovirus and then spreading all across the world is an example of the potential of the parvoviruses to cause pandemics [147]. Simian parvovirus, which causes severe anemia in cynomolgus macaques, has a close homology to human virus B19 and is a potential zoonosis [148,149].

#### 5.2.3. Polyomaviridae

Polyomaviruses are a family of viruses known to cause infections in immunocompromised patients [106]. Human JC (John Cunningham) polyomavirus (JCPyV), which causes progressive multifocal leukoencephalopathy, binds to α-2, 3- and α-2, 6-linked SAs [150,151]. Even the recently discovered human polyomavirus (HpyV9) was shown to preferentially bind to terminal Neu5Gc attached to lactosamine compounds with α-2, 3 linkage [152]. Polyomaviruses are associated with cancers in humans [153]. Recent investigations have suggested the identification of novel polyomaviruses from non-human primates to circulate among humans and a zoonosis possibility from bovine polyomaviruses following close human encounters [153,154].

## 6. Conclusions and Future Directions

**Figure 2:**
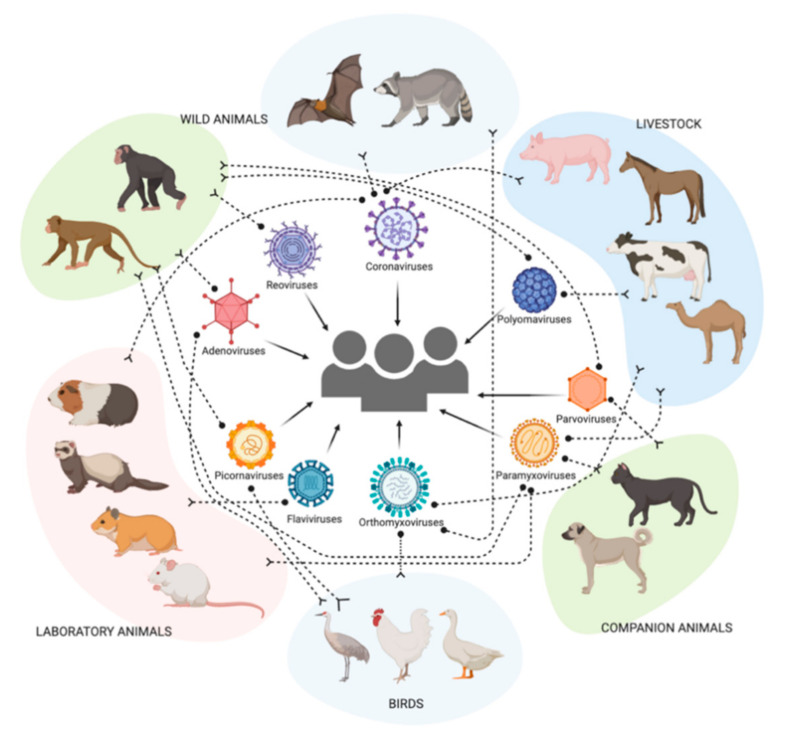
Extensive distribution of sialic acid receptors among various animal hosts can potentially facilitate the emergence of multiple viruses and facilitate zoonotic spillover, which can lead to epidemics and pandemics.

The spread of infections has been documented since time immemorial. Most viral pathogens in humans have animal origins and arose through cross-species transmission. Viral emergence will continue to be the biggest threat with thousands of viruses constantly mutating and evolving as they transmit between domestic and wild animals. Growing global human population and population shift due to urbanization have increased exposure of domestic and wild animals and humans. Consequently, the evolving human–animal–environment interactions have significantly increased chances of potential spillover of viruses from domestic and or wild animals to humans [155]. Emerging infections caused by Ebola, Nipah, Hendra, influenza, and coronaviruses in the highly populated regions of Asia and Africa have been evident in the last two decades [156,157]. A virus newly adapted to humans post a spillover event achieving a sustained human-to-human transmission is a rare occurrence. However, with increasing proximity between humans and wildlife reservoirs, the chances of such spillover events are extremely high (Figure 2).

A critical understanding of emerging viral infections with a multi-pronged approach is essential to prevent virus spillover. From a receptor standpoint, widespread presence of SA receptors in domestic, wild animals and humans that a particular virus can bind provides an opportunity to jump species and adapt to the human host.

There has been a significant progress in our understanding of entry receptors of many viruses and distribution of those receptors across animal species. As described in this review, understanding the structure and distribution of SA receptors in different hosts as well as characterizing their functional roles have allowed insights into the tropism, inter-species emergence, and pathogenesis of diverse group of zoonotic viruses. However, further studies to identify and characterize SA receptors among other animal species is important to investigate the range of viruses to which an animal host is naturally susceptible. This information will help develop more targeted and focused surveillance strategies for better detection of zoonotic virus spillover and prediction of their potential to cause epidemics and pandemics. In addition, a better understanding of the processes involved in the cellular entry of viruses will uncover new strategies for designing therapeutics and vaccines.

## Figures and Tables

**Table 1:** Distribution of sialic acid receptors in different animal species.

Host	Distribution of SA α2,6-Gal	Distribution of SA α2,3-Gal	References
Humans	Ciliated and non-ciliated cells in respiratory tract; ileal epithelium	Ciliated cells in bronchioles and alveoli; colon epithelium; endothelial cells of blood vessels and inflammatory cells	[22,30,36,38,39,40,41,56]
Non-human primates	Goblet cells of submucosal glands and submucosal connective tissue; ciliated cells on epithelium in URT	Goblet cells of submucosal glands and submucosal connective tissue; Type II pneumocytes in lungs	[22,37,41,42]
Swine	Ciliated epithelia and goblet cells in trachea and bronchus; alveolar epithelium; duodenum; colon	Alveolar epithelium; duodenum; colon	[31,43,44,45]
Equines	Along nasal mucosa to bronchus; goblet cells	Ciliated nasal mucosa; trachea; bronchus; goblet cells	[49]
Bovines	Deficient in trachea;	Trachea	[45,47,48,49]
Camelidae	Not reported	Nasal respiratory epithelium; alveolar epithelial cells	[50]
Canines	Goblet cells and sub-epithelial regions of nasal mucosa and trachea; lamina propria; large intestine	Nasal mucosa; trachea; bronchi; alveoli; goblet cells and sub-epithelial regions of nasal mucosa and trachea; large intestine	[51,52]
Felines	Ciliated pseudostratified epithelial cells; goblet cells; alveolar epithelial cells	Ciliated pseudostratified epithelial cells; goblet cells; sub-epithelial connective tissue; alveolar epithelial cells	[51,53]
Bats	Lamina propria; submucosa of trachea and bronchi; alveoli; goblet cells; serosa of intestine	Epithelial cells of trachea; mucosal lining of intestinal villi	[54,55]
Plateau Pika	Lamina propria and mucous glands of trachea; alveolar epithelial cells; duodenum; ileum; rectum	Lamina propria and mucous glands of trachea; alveolar epithelial cells; duodenum; ileum; rectum.	[57]
Raccoon	Epithelium and sub-epithelial regions in URT	Epithelial and sub-epithelial regions of trachea and bronchi.	[58]
Guinea pig	Epithelial cells of nasal and tracheal mucosa	Alveolar cells and vascular endothelial cells.	[59]
Ferrets	Ciliated cells and submucosal glands in trachea and bronchus; alveoli	Lamina propria and sub-mucosal areas; alveoli	[53,60,61]
Hamsters	Distal end of the nasal cavity; pharynx; trachea; bronchus	Proximal end of the nasal cavity; pharynx; trachea; bronchus; lungs	[62,63]
Mice	Sub-epithelial locations; trachea; bronchi; bronchioles; alveolar cells; intestines; lymphoid nodules of cecum	Sub-epithelial locations; trachea; bronchi; bronchioles; alveolar cells; intestines	[64]
Galliformes	Nasal cavity to lungs; tracheal epithelium of chickens and quails	Nasal cavity to lungs; trachea of ducks; intestines	[29,65,66]
Passeriformes	Epithelial cells of respiratory tract; sub-epithelial regions and glands	Epithelial cells of respiratory tract (predominant); sub-epithelial regions and glands; intestines	[21,66]
Columbiformes	Respiratory tract	Respiratory tract	
Anseriformes	Respiratory and intestinal tracts; lungs; ileal and cecal enterocytes; duodenum; jejunum	Respiratory and intestinal tracts; respiratory epithelial cells; ciliated cells; goblet cells; sub-epithelial regions of trachea and bronchi; goblet cells of small and large intestines	[21,66]
Charadriiformes	Respiratory tract epithelium; marginal in intestines	Respiratory tract epithelium	[21]
Gruiformes, Pelecaniformes, Gaviiformes, Ciconiiformes	Respiratory epithelium	Respiratory and intestinal epithelium	[21]

## Data Availability

Not applicable.
